# Integrating real-world data from Brazil and Pakistan into the OMOP common data model and standardized health analytics framework to characterize COVID-19 in the Global South

**DOI:** 10.1093/jamia/ocac180

**Published:** 2022-10-20

**Authors:** Elzo Pereira Pinto Junior, Priscilla Normando, Renzo Flores-Ortiz, Muhammad Usman Afzal, Muhammad Asaad Jamil, Sergio Fernandez Bertolin, Vinícius de Araújo Oliveira, Valentina Martufi, Fernanda de Sousa, Amir Bashir, Edward Burn, Maria Yury Ichihara, Maurício L Barreto, Talita Duarte Salles, Daniel Prieto-Alhambra, Haroon Hafeez, Sara Khalid

**Affiliations:** Center of Data and Knowledge Integration for Health (Cidacs), Fiocruz—Brazil, Parque Tecnológico da Edf, Tecnocentro, R. Mundo, Salvador, BA 41745-715, Brazil; Center of Data and Knowledge Integration for Health (Cidacs), Fiocruz—Brazil, Parque Tecnológico da Edf, Tecnocentro, R. Mundo, Salvador, BA 41745-715, Brazil; Center of Data and Knowledge Integration for Health (Cidacs), Fiocruz—Brazil, Parque Tecnológico da Edf, Tecnocentro, R. Mundo, Salvador, BA 41745-715, Brazil; Shaukat Khanum Memorial Cancer Hospital and Research Centre, Johar Town, Lahore, 54840, Pakistan; Shaukat Khanum Memorial Cancer Hospital and Research Centre, Johar Town, Lahore, 54840, Pakistan; Fundació Institut, Universitari per a la recerca a l’Atenció Primària de Salut Jordi Gol i Gurina (IDIAPJGol), Barcelona, 587 08007, Spain; Center of Data and Knowledge Integration for Health (Cidacs), Fiocruz—Brazil, Parque Tecnológico da Edf, Tecnocentro, R. Mundo, Salvador, BA 41745-715, Brazil; Center of Data and Knowledge Integration for Health (Cidacs), Fiocruz—Brazil, Parque Tecnológico da Edf, Tecnocentro, R. Mundo, Salvador, BA 41745-715, Brazil; Center of Data and Knowledge Integration for Health (Cidacs), Fiocruz—Brazil, Parque Tecnológico da Edf, Tecnocentro, R. Mundo, Salvador, BA 41745-715, Brazil; Shaukat Khanum Memorial Cancer Hospital and Research Centre, Johar Town, Lahore, 54840, Pakistan; Centre for Statistics in Medicine, Botnar Research Centre, University of Oxford, Oxford, OX3 7LD, United Kingdom; Center of Data and Knowledge Integration for Health (Cidacs), Fiocruz—Brazil, Parque Tecnológico da Edf, Tecnocentro, R. Mundo, Salvador, BA 41745-715, Brazil; Center of Data and Knowledge Integration for Health (Cidacs), Fiocruz—Brazil, Parque Tecnológico da Edf, Tecnocentro, R. Mundo, Salvador, BA 41745-715, Brazil; Fundació Institut, Universitari per a la recerca a l’Atenció Primària de Salut Jordi Gol i Gurina (IDIAPJGol), Barcelona, 587 08007, Spain; Centre for Statistics in Medicine, Botnar Research Centre, University of Oxford, Oxford, OX3 7LD, United Kingdom; Shaukat Khanum Memorial Cancer Hospital and Research Centre, Johar Town, Lahore, 54840, Pakistan; Centre for Statistics in Medicine, Botnar Research Centre, University of Oxford, Oxford, OX3 7LD, United Kingdom

**Keywords:** health equity, observational data, federated data network, COVID-19, OMOP common data model

## Abstract

**Objectives:**

The aim of this work is to demonstrate the use of a standardized health informatics framework to generate reliable and reproducible real-world evidence from Latin America and South Asia towards characterizing coronavirus disease 2019 (COVID-19) in the Global South.

**Materials and Methods:**

Patient-level COVID-19 records collected in a patient self-reported notification system, hospital in-patient and out-patient records, and community diagnostic labs were harmonized to the Observational Medical Outcomes Partnership common data model and analyzed using a federated network analytics framework. Clinical characteristics of individuals tested for, diagnosed with or tested positive for, hospitalized with, admitted to intensive care unit with, or dying with COVID-19 were estimated.

**Results:**

Two COVID-19 databases covering 8.3 million people from Pakistan and 2.6 million people from Bahia, Brazil were analyzed. 109 504 (Pakistan) and 921 (Brazil) medical concepts were harmonized to Observational Medical Outcomes Partnership common data model. In total, 341 505 (4.1%) people in the Pakistan dataset and 1 312 832 (49.2%) people in the Brazilian dataset were tested for COVID-19 between January 1, 2020 and April 20, 2022, with a median [IQR] age of 36 [25, 76] and 38 (27, 50); 40.3% and 56.5% were female in Pakistan and Brazil, respectively. 1.2% percent individuals in the Pakistan dataset had Afghan ethnicity. In Brazil, 52.3% had mixed ethnicity. In agreement with international findings, COVID-19 outcomes were more severe in men, elderly, and those with underlying health conditions.

**Conclusions:**

COVID-19 data from 2 large countries in the Global South were harmonized and analyzed using a standardized health informatics framework developed by an international community of health informaticians. This proof-of-concept study demonstrates a potential open science framework for global knowledge mobilization and clinical translation for timely response to healthcare needs in pandemics and beyond.

## Graphical Abstract

**Graphical Abstract:**
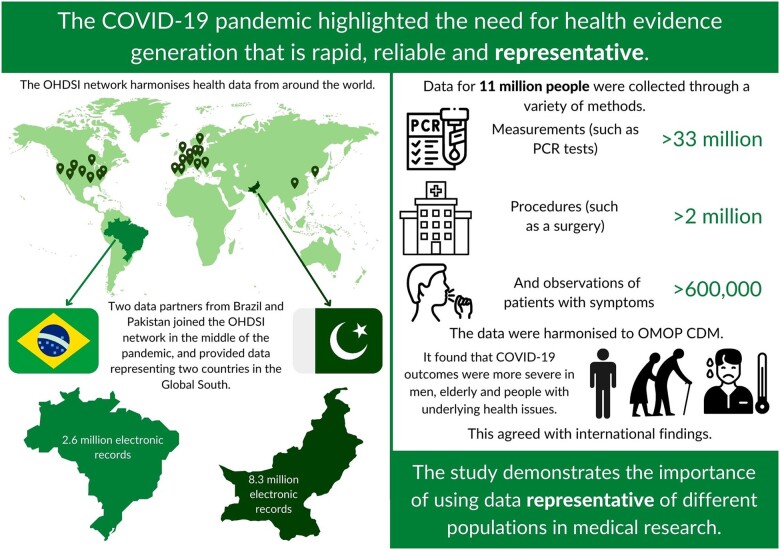

## INTRODUCTION

The coronavirus disease 2019 (COVID-19) pandemic placed an unprecedented burden on global healthcare systems, particularly in under-resourced communities. With a COVID-19 death rate of 289 people in every 100 000 in late 2021, Brazil was the third worst hit country in the world.^1^^,^^2^ South Asia is home to a quarter of the world’s population and was a COVID-19 hotspot: India had the second-highest caseload in the world and neighboring Pakistan the third-highest in Asia.^2^ South Asian ethnicity is associated with a high risk of severe COVID-19 and related mortality.^3^^,^^4^ However, there are little or no real-world COVID-19 data from South Asia or Brazil. As the pandemic continues, a full picture of COVID-19’s natural history, globally and in South Asia and Latin America, is needed.^5^

Routinely collected health data originate from a variety of real-world healthcare settings and are often not recorded for research use. Globally, and particularly in resource-limited settings, there is a lack of standardized systems for curating and analyzing these heterogenous data.^6^ Consequently it can be difficult to compare any resulting evidence, limiting the potential to impact health-care policies and interventions. There is therefore a need for data science ecosystems for data harmonization and related governance, standardized analytics and related capacity building, and evidence generation that is transparent, timely, and transportable across health settings.^7^

The health informatics community has begun using trusted research environments and federated distributed data networks (FDNs), motivated by the need for accelerated knowledge mobilization and clinical translation in the COVID-19 pandemic.^8–11^ The Observational Health Data Sciences and Informatics (OHDSI) collaboration^12^ has led to the development of an open source FDN framework.^13–18^ It enables mapping of participating data sources to the Observational Medical Outcomes Partnership (OMOP) common data model (CDM), standardized analytical open-source tools that data partners can run locally on their mapped data, and aggregation of site-specific results via open access. This strategy has gained credibility as a best-practice approach for conducting rapid, transparent, and reproducible international research.^8^ It has been leveraged to generate observational evidence for COVID-19 and has impacted international clinical guidelines and regulatory safety warnings.^17^^,^^19–34^ However, health data sources and data partners from low- and middle-income country (LMIC) settings remain largely underrepresented in such endeavors.

In this article, we describe the harmonization of 2 health databases from Brazil and Pakistan to the OMOP CDM. We illustrate their use for describing COVID-19 patient characteristics in these 2 large Global South countries. The ultimate aim of this work is to demonstrate the implementation of an international distributed network analytics approach to accelerate the clinical translation and global knowledge mobilization.

### What this study adds

The international OHDSI COVID-19 collaboration previously harmonized data from >500 million people, including >7 million people tested for COVID-19 and >1.2 million with COVID-19, from 16 databases in the United States, Europe, China, and South Korea, resulting in one of the largest multinational characterization studies to understand covariates, treatments, and outcomes related to COVID-19.^27^ Latin America and South Asia together represent a third of the world’s population but could not be included in the international efforts due to a lack of reliable data and health informatics infrastructure. This study adds, for the first time, a large Pakistani database from the Shaukat Khanum Memorial Cancer Hospital and Research Centre (SKMCH&RC) and Brazilian Health Surveillance Service Data for State of Bahia (Center for Health Data and Knowledge Integration CIDACS/IGM/FIOCRUZ) to the OHDSI-OMOP data network (Supplementary Figure S1). To our knowledge, these are the first OMOP-harmonized real-world datasets representating ethnically diverse populations in Latin America and South Asia.

## MATERIALS AND METHODS

### Overview of the Federated Data Network

**Figure 1.**
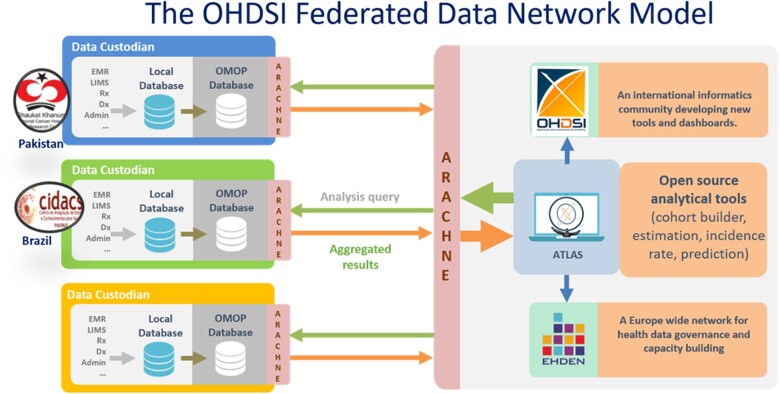
Schematic structure of the federated data network developed by the Observational Health Data Sciences and Informatics (OHDSI) community for standardized health analytics using the OMOP common data model (CDM). OMOP: Observational Medical Outcomes Partnership.

This work adopted a distributed FDN framework designed for rapid and reproducible research and knowledge exchange, using the OMOP CDM (Figure 1). The OMOP CDM has been developed to work with a wide range of routinely collected health-care data;^14–16^ numerous databases from North America, Europe, and beyond have been mapped to it.^24^^,^^25^^,^^28^^,^^29^ The OMOP CDM has also been used to inform several studies relating to the COVID-19 pandemic.^17^^,^^19–34^ The FDN design allows for accelerated analytics with the same analysis code being run by each data partner and aggregated results shared, without any need to share patient-level data between data partners.

### OMOP mapping: extract, transform, and load

OMOP is an open-source CDM standard for harmonizing the structure and semantic representation of observational data.^16^ It follows a person-level relational database design to facilitate analysis of longitudinal person-level data such as clinical (eg, symptoms, diagnoses, drugs, procedures, devices, measurements, and text notes) and health system data (eg, healthcare provider, care site, and costs) that are organized into a set of predefined tables.^9^ The use of the OMOP CDM by participating researchers enables studies to be consistently developed, executed, and replicated across collaborator sites. The source data are extracted, transformed, and loaded (ETL) to map or conform it to the OMOP CDM.^16^ Over 3000 quality control checks on plausiblity, conformance, and completeness assess whether the mapped database is fit for use. Any errors identified during quality control are addressed by updating the ETL where possible.^16^ A summary of OHDSI tools used for OMOP mapping is presented in Supplementary Appendix SA; full details can be found in Ref.^16^

### Pakistan database

SKMCH&RC (www.shaukatkhanum.org.pk) is a 195-bed secondary and tertiary care hospital network in Pakistan that provides cancer and noncancer care and acts as a regional hub for COVID-19 cases. Its hospital information system contains electronic medical records for over 8.3 million people (52.7% female). This includes de-identified patient-level data on sociodemographics, laboratory results, clinical diagnoses (from on-site and community diagnostic laboratories), outcomes, prescriptions, hospital in-patient procedures, and mortality from December 1994 to present (June 1, 2022). All COVID-19 records to date have been mapped to the OMOP-CDM). The mapped dataset is hereafter referred to as the SKMCH&RC COVID-19 database.

### Brazil database

CIDACS-FIOCRUZ (www.cidacs.bahia.fiocruz.br) is a center for big health data linkage in Brazil. After the onset of the COVID-19 pandemic, CIDACS-FIOCRUZ developed a COVID-19 data integrated platform that contains aggregate and individual-level socioeconomic and demographic indicators extracted from the COVID-19 surveillance database for the State of Bahia, which covers a population of 15 million people (Supplementary Figure S5). It includes data on patient self-reported compulsory notifications of severe cases, hospitalizations, and deaths due to COVID-19 (SRAG), mild and moderate cases (ESUS), laboratory data (GAL), and vaccination data (VAC). This linked dataset, hereafter referred to as the CIDACS-FIOCRUZ COVID-19 database, contains 7 585 719 observations with data on age, sex, ethnicity, symptoms, outcome of suspected cases (hospitalization, intensive care unit [ICU] admission, death, and use of mechanical ventilator), observation period, and comorbidities at the time of notification.

### Characterization of COVID-19

#### Study settings

##### Participants

All individuals who were tested for COVID-19 on or after January 1, 2020 (Brazil) and on or after March 1, 2020 (Pakistan) until April 30, 2022 were included.

##### Study cohorts

Five COVID-19-related cohorts were considered:

1. those in the general population tested for COVID-19,
2. those who tested positive for or were diagnosed with COVID-19,
3. those hospitalized with COVID-19, within 30 days of a positive test or diagnosis of COVID-19,
4. those admitted to ICU with COVID-19, within 30 days of a positive test or diagnosis of COVID-19,
5. those who died with COVID-19 within 30 days of positive test or diagnosis of COVID-19.

The cohorts were not mutually exclusive. Detailed cohort definitions may be found in Supplementary Appendix SA.

##### Baseline characteristics

Sociodemographics (age, sex, and ethnicity) and medical history (body mass index [BMI], smoking status, and available comorbidities) were included.

### Statistical analysis

The baseline characteristics of the participants in each of the study cohorts were calculated for the Pakistan and Brazil COVID-19 cohorts, with counts and percentages for categorical variables and median and interquartile ranges (IQR) for continuous variables. For variables with missing data, estimates were based on cases without missingness (complete-case). BMI was not available in Pakistan data, whereas smoking status was not available in Brazil data. To plot the distribution of COVID-19 cases over time, the number of cases per month was calculated for each cohort, counted at the time an individual entered a given cohort.

The Brazil and Pakistan COVID-19 datasets were analyzed independently and simultaneously. At the time of writing, all COVID-19 concepts from the Brazilian CIDACS-FIOCRUZ COVID-19 database had been mapped to the OMOP CDM via ETL implementation, however, mapping of patient-level records was yet to be done. Baseline characterization was therefore based on the source CIDACS-FIOCRUZ database. In contrast as the Pakistani SKMCH&RC COVID-19 database was fully mapped to the OMOP CDM, analyses were conducted using its CDM version.

## RESULTS

### Harmonization to OMOP CDM

A total of 109 504 medical concepts in the SKMCH&RC COVID-19 dataset were mapped from the source database to 108 684 matching concepts in the OMOP CDM (summarized in Table 1). In the CIDACS-FIOCRUZ COVID-19 dataset, 921 concepts were mapped to 915 matching OMOP concepts (summarized in Table 2). This concept mapping allowed source concepts from hundreds of tables to be matched to a universal set of 8 domains in the OMOP CDM: “Provider”, “Measurement”, “Specimen”, “Procedure”, “Device”, “Drug Exposure”, “Condition”, and “Unit of Measurement” (Tables 1 and 2). For example, the SKMCH&RC COVID-19 CDM included 33.9 million laboratory results (“Measurement” table) for 349 879 patient records (“Person” table) collected from 357 sites around the country (“Location” table) as shown in Supplementary Table S1. Although most of the concepts in the source domains had matching concepts in the corresponding CDM domains, 820 (0.7%) Pakistani concepts and 6 (0.6%) Brazilian concepts did not have matching concepts and were not mapped. The 820 unmapped concepts in Table 1 correspond to 6.46%, 0.06%, and 9.96% of the “Drug exposure,” “Measurement,” and “Procedure” domains, respectively.

**Table 1.** Summary of SKMCH&RC COVID-19 (Pakistan) database concepts mapped to the OMOP CDM

Source domain	CDM domain	Number of source concepts	Number of mapped concepts	Number of unmapped concepts
Doctor specialty	Provider	57	57	0
Pathology test	Measurement	591	591	0
Pathology specimen	Specimen	484	465	19
Pathology text results	Measurement	1028	758	270
Procedures	Procedure	4986	4977	9
Surgical supplies	Device	1314	1281	33
Vital signs	Measurement	14	14	0
Drug route	Drug exposure	31	30	1
Drug generics	Drug exposure	2643	2299	344
ICD codes	Condition	97 808	97 808	0
Unit of measurement	Used in multiple domains	548	404	144
Total		109 504	108 684	820

CDM: common data model; OMOP: Observational Medical Outcomes Partnership.

**Table 2.** Summary of CIDACS-FIOCRUZ COVID-19 (Brazil) database concepts mapped to the OMOP CDM

Source domain	Domain	Number of source concepts	Number of mapped concepts	Number of unmapped concepts
Epidemiological clinical data	Condition	31	31	0
Epidemiological clinical data	Drugs	0	0	0
Epidemiological clinical data	Devices	0	0	0
Epidemiological clinical data	Procedures	33	33	0
Epidemiological clinical data	Observations	56	56	0
Epidemiological clinical data	Measurements	34	34	0
Identification	Ethnicity/race	313	307	6
Identification	Gender	2	2	0
Localization	Visit	7	7	0
Localization	Geography	444	444	0
Total	Total	921	915	6

CDM: common data model; OMOP: Observational Medical Outcomes Partnership.

Supplementary Figures S2 and S3 summarize the ETL stages of the harmonisation process for the SKMCH&RC (Pakistan) and CIDACS-FIOCRUZ (Brazil) COVID-19 datasets, respectively. The steps to translate or map source data to OMOP CDM were customized for each database. As the SKMCH&RC COVID-19 database was available as a pre-existing electronic health record database generated by SKMCH&RC’s hospital information system, ETL was implemented directly (see figure for example of ETL code mapping). Before ETL could be applied to the CIDACS-FIOCRUZ database, relevant COVID-19 fields had to first be extracted from 2 heterogeneous source datasets (ESUS and SRAG).

Supplementary Figure S4 shows the results for 3486 data quality checks performed on the SKMHR&C COVID-19 database within the FDN framework for assessment of the ETL process. A total of 12 errors were found in the type “measurement unit not found” and “unmapped concept” with an overall pass rate of 100% (with regards to plausibility, conformance, and completeness) these errors did not impact the study.

**Figure 2.**
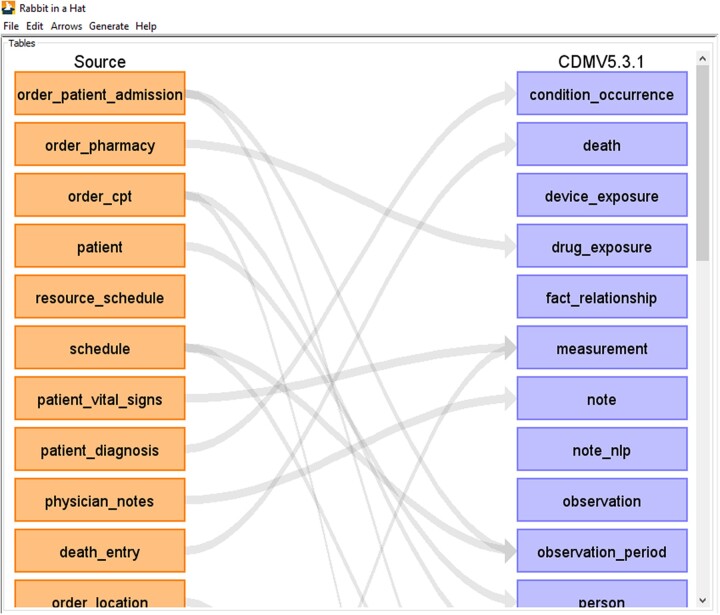
An example of concept mapping during the extract—transform—load (ETL) process for the Pakistan database using the Rabbit in a Hat tool^16^ available within the OHDSI analytical pipeline.

Figure 2 shows an example of the ETL concept mapping process for the Pakistan database.

### COVID-19 characterization

#### Pakistan

**Figure 3.**
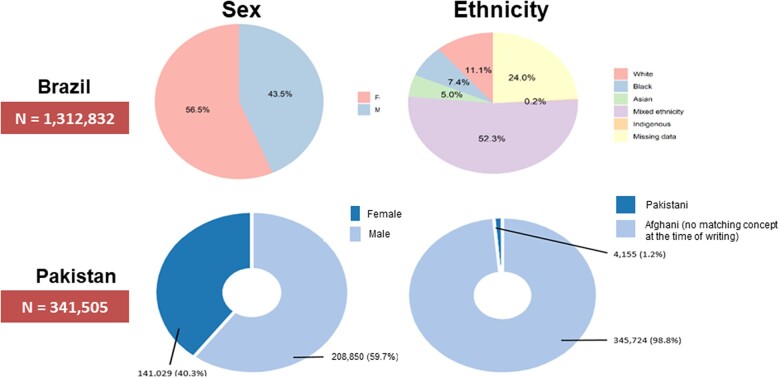
Distribution of sex (left) and ethnicity (right) in the general population tested for COVID-19, in the Pakistan (top) and Brazil (bottom) datasets. A matching concept for ethnicity/race = “Afghan” was not available in the OMOP CDM at the time of writing. The 1.2% of Afghan individuals in the Pakistan database are therefore labeled as “no matching concept.” CDM: common data model; COVID-19: coronavirus disease 2019; OMOP: Observational Medical Outcomes Partnership.

The SKMCH&RC database contained information on a total of 8 334 767 unique individuals, of whom 341 505 were tested for COVID-19 between March 1, 2020 and April 30, 2022. Table 3 summarizes the baseline characteristics of people who were tested, tested positive, were hospitalized, were admitted to ICU, and who died. Figure 3 shows the distribution of sex and ethnicity in the SKMCH&RC database. 1.2% percent were of Afghan ethnicity; here labeled as “no matching concept” as the Afghan ethnicity concept was not available in the OMOP CDM at the time of writing.

**Table 3.** Baseline characteristics of the COVID-19 study cohorts (PAKISTAN)

Variable	Tested population (ie, general population)	Outpatient COVID-19 diagnosis or positive test	Hospitalized with COVID-19	ICU admission with COVID-19	COVID-19 death
*N*	341 505	88 771	447	33	117
Age (median [IQR])	36 [25–76]	40 [25–76]	48 [33–64]	52 [22–63]	53 [41–65]
Age group (*n* [%])					
Under 20	35 090 (10.3)	6453 (7.3)	68 (15.2)	3 (9.1)	10 (8.6)
20–29	72 958 (21.4)	16 064 (18.1)	39 (8.7)	3 (9.1)	3 (2.6)
30–39	86 984 (25.5)	20 748 (23.4)	61 (13.7)	6 (18.2)	14 (12.0)
40–49	57 221 (16.8)	14 931 (16.8)	73 (16.3)	1 (3.0)	20 (17.0)
50–59	42 592 (12.5)	13 284 (15.0)	93 (20.8)	10 (10.3)	25 (21.4)
60–69	29 678 (8.1)	10 620 (12.0)	81 (18.1)	9 (27.3)	28 (24.0)
70–79	13 227 (3.9)	5137 (5.8)	27 (6.0)	1 (3.0)	12 (10.3)
80 or older	3755 (1.1)	1534 (1.7)	5 (1.1)	0 (0)	4 (3.4)
Sex: male (n [%])	204 125 (59.7)	50 964 (54.4)	239 (53.5)	17 (51.5)	64 (54.7)
Smoking status (*n* [%])					
Current smoker	197 (0.06)	49 (0.06)	14 (3.13)	0 (0)	3 (2.56)
Ex-smoker	414 (0.12)	99 (0.11)	26 (5.82)	2 (6.06)	6 (5.13)
Nonsmoker	4225 (1.24)	1040 (1.17)	249 (55.70)	14 (42.42)	56 (47.86)
*Missing*	*336 669 (98.5)*	*87 583(98.6)*	*158(35.4)*	*17(51.5)*	*52 (44.44)*
BMI (median [IQR])	NA	NA	NA	NA	NA
Comorbidities (*n* [%])					
Diabetes mellitus	3432 (1)	1155 (1.30)	76 (17)	12 (36.36)	27 (23.08)
Heart disease	743 (0.22)	243 (0.27)	19 (4.25)	4 (12.12)	7 (5.98)
Hypertensive disorder	4525 (1.33)	1388 (1.56)	100 (22.37)	12 (36.36)	31 (26.50)
Renal impairment	973 (0.28)	207 (0.23)	82 (18.34)	13 (39.39)	38 (32.48)
Cancer	5868 (1.02)	1321 (1.49)	314 (70.25)	21 (63.64)	66 (56.41)
Dementia	6 (0)	2 (0)	1 (0.22)	0 (0)	1 (0.85)
Autoimmune disease	1 (0)	1 (0)	0 (0)	0 (0)	0 (0)
COPD	57 (0.02)	14 (0.02)	5 (1.12)	2 (6.06)	3 (2.56)
Asthma	316 (0.09)	109 (0.12)	13 (2.90)	0 (0)	3 (2.56)
Obesity	NA	NA	NA	NA	NA

BMI: body mass index; ICU: intensive care unit; IQR: interquartile range; COVID-19: coronavirus disease 2019.

**Figure 4.**
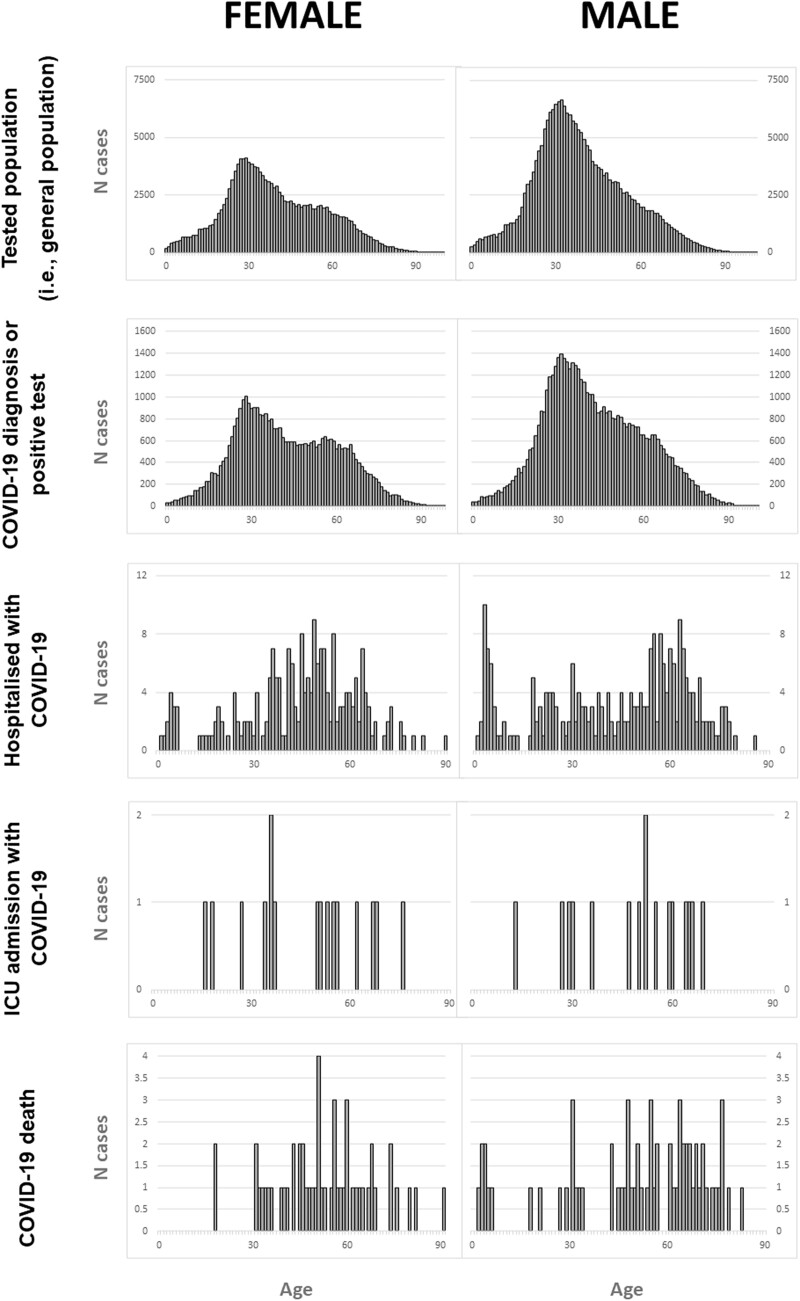
Distribution of age by sex in each COVID-19 cohort (PAKISTAN). COVID-19: coronavirus disease 2019.

In total, 88 771 (26%) of those tested had a COVID-19 diagnosis or positive test result. A greater proportion of male participants were tested (59.7%), tested positive (54.4%), hospitalized (53.5%), admitted to ICU (51.5%), or died (54.7%), compared with female participants. The average (median [IQR]) age of those tested in the general population was 36 years [25–76], whereas those who died (53 [41–65]), were admitted to ICU (52 [22–63]), or were hospitalized (48 [33–64]) were older, for both men and women (Figure 4). Comorbidities followed a similar trend with diabetes, hypertensive disorder, and renal impairment in 23.08%, 26.5%, and 32.48% of those who died in hospital compared with 1%, 1.33%, and 0.28% of the same in the general tested population. Figure 6 shows the distribution of COVID-19 cases over time.

#### Brazil

The CIDACS-FIOCRUZ database contained information on 2 669 866 unique individuals from the general population, of whom 1 312 832 (49.2%) met the inclusion criteria and had a valid COVID-19 test. In total, 752 699 (57.3%) tested positive between January 1, 2020 and April 30, 2022. In those tested, 56.5% were female; 52.3% had mixed, 11.1% White, 7.4% Black, 5% Asian, and 0.2% Indigenous ethnicity; ethnicity was missing for 24% (Figure 3).

**Figure 5.**
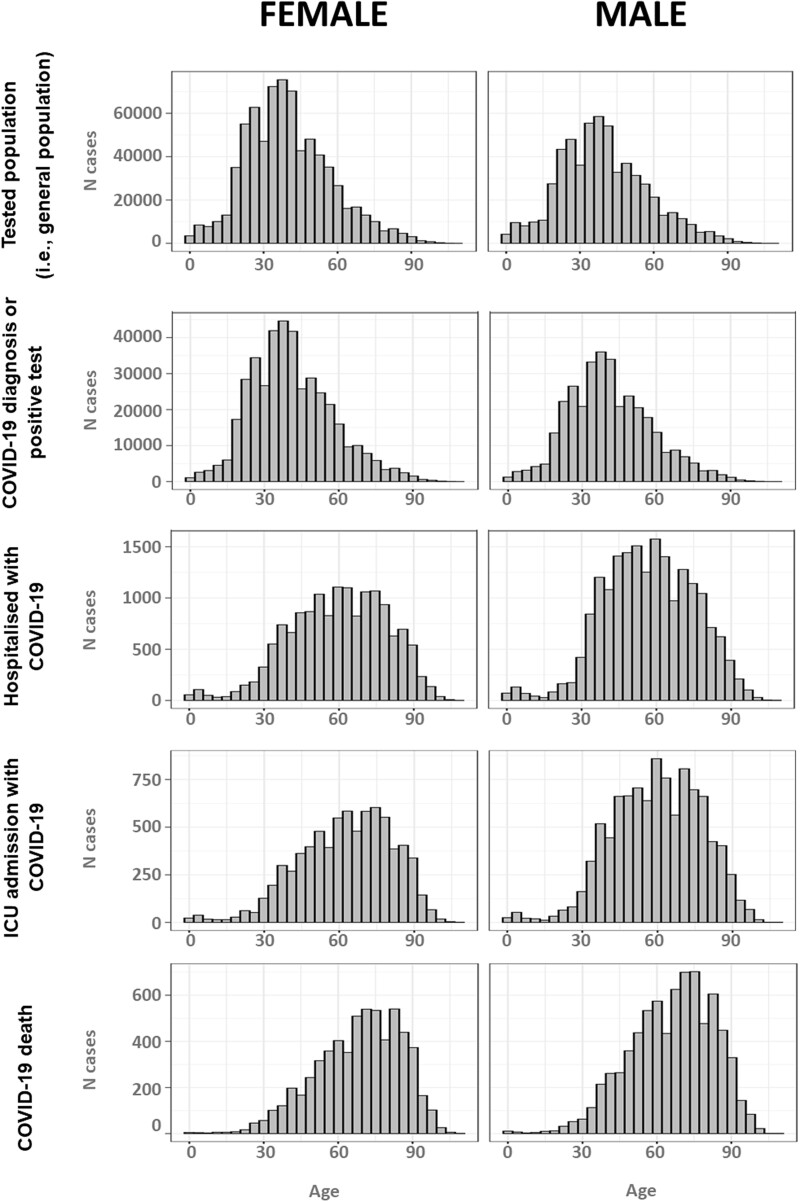
Distribution of age by sex in each COVID-19 cohort (BRAZIL). COVID-19: coronavirus disease 2019.

Table 4 summarizes the baseline characteristics of those who were tested, tested positive, hospitalized, admitted to ICU, and those who died. A smaller proportion of male participants were tested (43.5%) and tested positive for COVID-19 (45%), whereas a greater proportion of male participants were hospitalized (57%), admitted to ICU (57%), or died (55%), compared with female participants. The average (median [IQR]) age of those tested in the general population was 38 years [27–50], whereas those who died (69 years [56–80]), were admitted to ICU (62 years [48–75]), or were hospitalized (58 years [45–72]) were much older, for both men and women (Figure 5). Comorbidities followed a similar trend. For example, 28% of those hospitalized, 33% of those admitted to ICU, and 32% of those who died had diabetes, compared with 3.6% in the general population and 4% of those who tested positive. Heart disease was present in 48% of those hospitalized, 54% of those admitted to ICU, and 50% of those who died, compared with 5.8% of the general population and 6.5% of those who tested positive.

**Table 4.** Baseline characteristics of the COVID-19 study cohorts (BRAZIL)

Variable	Tested population (ie, general population)	Outpatient COVID-19 diagnosis or positive test	Hospitalized with COVID-19	ICU admission with COVID-19	COVID-19 death
*N*	1 312 832	752 699	34 699	17 041	13 877
Age (median [IQR])	38 [27–50]	39 [29–51]	58 [45–72]	62 [48–75]	69 [56–80]
Age group (*n* [%])					
Under 20	127 765 (9.7)	54 277 (7.2)	745 (2.1)	271 (1.6)	77 (0.6)
20–29	257 065 (20)	137 443 (18)	1044 (3.0)	376 (2.2)	190 (1.4)
30–39	317 037 (24)	187 526 (25)	3809 (11)	1484 (8.7)	649 (4.7)
40–49	264 994 (20)	162 490 (22)	5791 (17)	2472 (15)	1374 (9.9)
50–59	167 933 (13)	105 102 (14)	6653 (19)	3118 (18)	2075 (15)
60–69	94 587 (7.2)	57 971 (7.7)	6276 (18)	3339 (20)	2735 (20)
70–79	51 002 (3.9)	30 199 (4.0)	5451 (16)	3142 (18)	3138 (23)
80 or older	32 449 (2.5)	17 691 (2.4)	4930 (14)	2839 (17)	3639 (26)
Sex: male (*n* [%])	580 058 (43.5)	338 190 (45)	19 608 (57)	9755 (57)	7699 (55)
BMI (median [IQR])	32 [30–36]	32 [30–36]	33 [30–36]	32 [30–36]	33 [29–35]
*Missing (n)*	1 312 077	752 045	34 066	16 693	13 743
Comorbidities (*n* [%]0					
Diabetes mellitus	46 944 (3.6)	30 368 (4.0)	9644 (28)	5573 (33)	4494 (32)
Heart disease	76 191 (5.8)	48 762 (6.5)	16 537 (48)	9260 (54)	6904 (50)
Renal impairment	6585 (0.5)	4041 (0.5)	1647 (4.7)	1148 (6.7)	977 (7.0)
Cancer	665 (<0.1)	464 (<0.1)	353 (1.0)	203 (1.2)	225 (1.6)
Dementia	415 (<0.1)	243 (<0.1)	199 (0.6)	111 (0.7)	120 (0.9)
Autoimmune disease	10 035 (0.8)	5374 (0.7)	1369 (3.9)	721 (4.2)	769 (5.5)
Respiratory disease	23 618 (1.8)	12 478 (1.7)	2169 (6.3)	1224 (7.2)	1013 (7.3)
Obesity	14 440 (1.1)	10 386 (1.4)	4075 (12)	2429 (14)	1348 (9.7)

BMI: body mass index; ICU: intensive care unit; IQR: interquartile range; COVID-19: coronavirus disease 2019.

**Figure 6.**
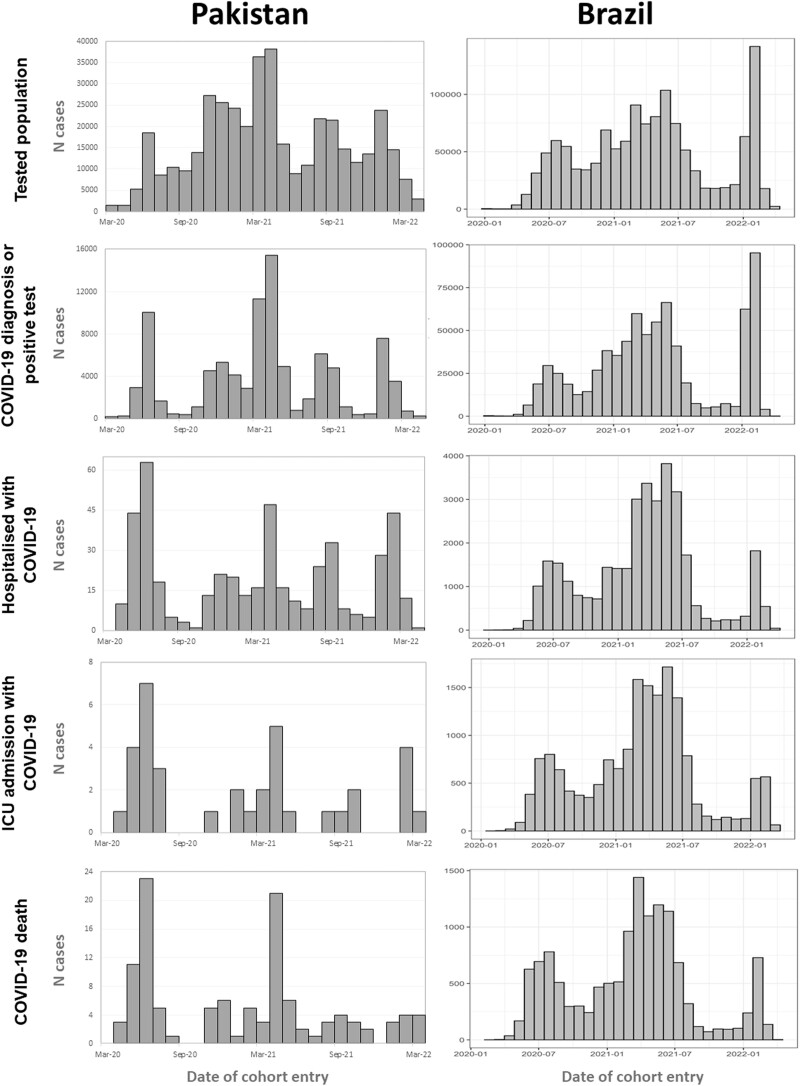
Distribution of cases over time for each COVID-19 cohort. COVID-19: coronavirus disease 2019.

Figure 6 shows the number of cases over calendar time, stratified by age, illustrating COVID-19 waves in Brazil and Pakistan over the course of the pandemic. Although testing peaked in 2022, the average number of COVID-19 hospitalizations, ICU admissions, and deaths was smaller in 2022 than in 2020/2021 in both countries.

## DISCUSSION

The COVID-19 pandemic highlighted health disparities and the need for globally accessible healthcare solutions that are equitable, timely, and impactful. This is turn necessitates an ecosystem of rapid, reproducible, and reliable evidence generation. There is a growing body of evidence to suggest that such translational research may be possible with heterogeneous yet harmonized, quality-controlled, well-governed real-world health data using standardised approaches such as distributed federated data networks.^17^^,^^19–34^ We applied one such framework (OMOP) developed by a global health informatics community (OHDSI) to health data from 2 geographically and sociodemographically diverse databases. Key pillars of the OMOP framework were leveraged namely data governance, data harmonization, and standardized analytics for transparent and fair health informatics.

### Harmonization to OMOP CDM — Insights

COVID-19 data from Brazil and Pakistan collected from and representative of different health-care settings were mapped to the OMOP CDM. The data from Brazil were generated from a bespoke patient-reported COVID-19 notification system that was developed for surveillance purposes and later linked with hospitalization and vaccination records from one state within Brazil. The data from Pakistan were extracted from an existing hospital information system spread across Pakistan. Despite the heterogeneity in the source data, it was possible to harmonize the data to a universal data vocabulary set. The ETL process for mapping to OMOP was tailored to each source database, to deal with their differing levels of complexity.

The Brazilian dataset was comparatively more complex and the mapping process correspondingly more time-consuming than the Pakistan dataset (Supplementary Figure S6). It involved the linkage of 181 tables retrieved from separate administrative datasets (SRAG, ESUS, GAL, and VAC) of the Brazilian Ministry of Health COVID-19 Surveillance system, which in itself was developed through a modification of the Brazil Influenza Surveillance System for pandemic response. In addition, there was not a unique key to merge the separate datasets. A deterministic linkage algorithm had to be derived using a person identification variable (comprising 5 identifiers), common sociodemographic variables such as age, sex, and municipality of residence. These variables are recorded under different names in the separate datasets with differing degrees of completeness. A key lesson learnt therefore was the impact of data complexity on speed and scale of research.

Several generalizable insights may also be drawn from this work. When mapping routinely collected datasets to a common model such as OMOP CDM, it is necessary to conduct feasibility of datasets suitable for real-world evidence generation, assessment of variables to be used for linkage, and determination of validated linkage algorithms if required.

Inevitably routine data may suffer from incompleteness. Data sources must therefore be sufficiently sized to maintain the ability to generate reliable knowledge. For instance, the data of birth was missing from 750 individuals in the Pakistan data; therefore, their records could not be included in the analysis.

Another key learning was the value of capacity building. A successful health informatics ecosystem depends on cross-collaboration between clinicians, data scientists, researchers, IT specialists, and information governance experts. Ultimately, a number of training needs were identified in order to build capacity for North-South research. For example, this study resulted in a real-world data science knowledge exchange programme between the research teams in Brazil, Pakistan, Spain, and the UK.

### COVID-19 characterization — Insights

Patient characteristics demonstrated the richness of data with respect to sociodemographic and clinical information. The Brazil database included individuals with Asian, Black, Indigenous, Mixed, and White ethnicities. Although most of the people in the Pakistan database were of Pakistani ethnicity, around 1.2% had Afghan ethnicity. To our knowledge, this is the first record of Afghan ethnicity in a research-ready electronic health records database.

We found that more men, older people, and people with underlying health conditions were hospitalized, admitted to ICU, or died due to COVID-19 than women, younger people, and people without underlying health issues in Brazil and Pakistan. This characterization of COVID-19 patients in Brazil and Pakistan agreed with previous findings from international settings.^24^^,^^29^^,^^33^^,^^35^^,^^36^ In particular, the largest international COVID-19 distributed network study to date (CHARYBDIS)^33^ found a similar trend by examining >22 000 patient characteristics from 4.5 million individuals from the United States, Europe (the Netherlands, Spain, the UK, Germany, France, and Italy), and Asia (South Korea and China). As the present study, CHARYBDIS reported worse outcomes in men, elderly people, and those with comorbidities, the most common being type 2 diabetes, hypertension, chronic kidney disease, and heart disease.

Although together the 2 databases contributed data on 11 million individuals from South Asia and Latin America, the data cannot be considered as fully representative. The Brazil database for example contained individuals from one state of Brazil (Bahia). The Pakistan database contained individuals from all over Pakistan, however, only those who sought healthcare within the SKMHR&C hospital network, via one of 2 pathways: (1) tested for COVID-19 in SKMHR&C hospital and admitted for COVID-19 or cancer care and (2) tested for COVID-19 in an SKMRHR&C community or on-site diagnostic lab but not admitted to the hospital. For the latter group, although complete COVID-19 diagnostic data were available, data capture on medical history was limited, potentially explaining the dominance of cancer as the main comorbidity in the characterization.

As with any routinely collected data not collected for health research by design, some of the information was incomplete. There were differences in the data capture and coverage from both settings; which in turn reflects the heterogeneity of the underlying settings in which the data originate, one being secondary care data and the other population-based surveillance data. For example ethnicity was missing in nearly a quarter of the individuals from Brazil. BMI was recorded for Brazilian individuals but not for Pakistan individuals, and vice versa for smoking. While data harmonization can improve usability and comparability of available data, the need for better collection at source remains.

Through this work, the 2 databases joined a growing health informatics community of over 100 international observational OMOP-mapped databases. By “speaking the same language” afforded by common data models such as OMOP, they can be used together to address critical health questions and generate both locally and globally relevant knowledge. Once data are mapped to a common data model, data partners in the FDN can run standardized analytical packages on their databases and contribute the results without needing to share patient-level data.

One of the key merits of the FDN framework approach is geographical and clinical scalability. Health data from anywhere in the world may be mapped to the OMOP CDM. Once harmonized, the data can support any clinical research through a standardized analytical pipeline that offers existing tools for causal inference, estimation, and prediction, for example. The FDN used here has underlying data governance, open science, and capacity building mechanisms, which make it well-suited to pandemic preparedness and response. As a result, it has been applied extensively in the COVID-19 response, including guidelines on COVID-19 drug safety and vaccine safety.^17^^,^^19–34^ Such an approach could be particularly well suited to LMIC settings as re-use of existing data can provide a cheaper alternative to or complement randomized clinical trials, which are generally time-consuming and expensive. It may also contribute to moving away from health research silos.

## CONCLUSION

This paper describes the process of mapping two health databases from Latin America and South Asia to the OMOP CDM for COVID-19 characterization. Future work includes scalability and capacity-building. This study is hoped to contribute to an ecosystem for observational evidence generation in 2 large regions in Latin America and South Asia to inform health interventions and policymaking for and beyond the COVID-19 pandemic.

## FUNDING

This publication is based on research funded in part by the Bill & Melinda Gates Foundation. The findings and conclusions contained within are those of the authors and do not necessarily reflect positions or policies of the Bill & Melinda Gates Foundation.

## AUTHOR CONTRIBUTIONS

All authors contributed equally to this work in accordance with ICMJE criteria for authorship; PN, EP, HH, DPA, TDS, MB, and SK were responsible for study conception and design; EP, PN, RFO, MUA, MAJ, SF, VAO, VF, FS, AB, EB, MYI, VM, and SK were involved in data curation, analysis, and interpretation; EP, PN, RFO, MUA, MAJ, SF, VAO, VF, FS, AB, EB, MYI, VM, DPA, and SK contributed to writing, reviewing and approving this manuscript, and are accountable for this work. SK has overall responsibility for this work.

## SUPPLEMENTARY MATERIAL

Supplementary material is available at *Journal of the American Medical Informatics Association* online.

## Supplementary Material

ocac180_Supplementary_Data

## Data Availability

All study documentations, including the study protocol and R code, are publicly available on request via GitHub (https://github.com/ohdsi-studies). Results for each of the databases participating in the study can be combined in an R Shiny application and then uploaded to the publicly available OHDSI Viewer Dashboard. The OHDSI tools involved in the prediction pipeline are regularly updated and revised versions are maintained on the GitHub. The OHDSI Forum is open for all to join, contribute to the development and use of tools, and cocreate scientific questions.
