# Supplementary material for: Integrating real-world data from Brazil and Pakistan into the OMOP common data model and standardized health analytics framework to characterize COVID-19 in the Global South
Source: J Am Med Inform Assoc. 2022 Oct 20;30(4):643–55. doi: 10.1093/jamia/ocac180 (PMC9619798; doi:10.1093/jamia/ocac180)
Supplement: ocac180_Supplementary_Data [file ocac180_supplementary_data.pdf]

Appendix A

Cohort Definitions

Unless specified, the same definition was used in Brazil and Pakistan data.

- General Population tested for COVID-19 – All patients who had a COVID-19 test (as defined below) irrespective of the result i.e. Detected, Not Detected.
- COVID 19 tested positive:
    - Pakistan: Lab Test "PCR Severe acute respiratory syndrome coronavirus 2 (SARS-CoV-2)"
    - Brazil: COVID 19 was defined as a positive RT-PCR or rapid antigen test.
- COVID 19 ICU admission: an ICU admission record with a record of a positive RT-PCR or rapid antigen test result in the 0 to 30 days prior to date of ICU admission.
    - Brazil: Covid-19 hospitalization cases are reported on admission, any subsequent usage of ICU in the same hospitalization is considered.
- COVID 19 Death: COVID 19 death was defined as a death record with a record of a positive RT-PCR or rapid antigen test result in the 0 to 30 days prior to date of death.
    - Pakistan: Death was considered if reported during a COVID-19 case hospitalisation;
    - Brazil: Death were considered if reported during a Covid-19 case hospitalization (SIVEP-Gripe_ or by municipal health authorities using morgue´s data (e-SUS VE)

OHDSI Tools

Below is a summary of OHDSI tools used in the study.

**Tool**	**Description [16]**	**How to Use**
**White Rabbit**	Performs a scan of the source data, providing detailed information on the tables, fields, and values that appear in a field. This scan will generate a report that can be used as a reference when designing the ETL, for instance when using the Rabbit-In-a-Hat tool.	https://www.ohdsi.org/analytic-tools/whiterabbit-for-etl-design/

**Rabbit in a Hat**	Rabbit-In-a-Hat uses the scan document and displays source data information through a graphical user interface to allow a user to connect source data structure to the CDM data structure.	https://www.ohdsi.org/analytic-tools/whiterabbit-for-etl-design/
**USAGI**	Mapping codes from a source system to the Observational Medical Outcomes Partnership (OMOP) Vocabulary.	www.ohdsi.org/web/wiki
**Athena**	A Web application for distributing and browsing the Standardized Vocabularies for all instances of an OMOP CDM	https://athena.ohdsi.org/search-terms/start
**Data Quality Dashboard**	After mapping has been performed, the Data Quality Dashboard applies a Harmonized Data Quality Assessment Terminology to the OMOP mapped data test plausibility, completeness, and conformance.	https://data.ohdsi.org/DataQualityDashboard/

Supplementary Table S1: Summary of the number of records from the source database included in various tables of the OMOP CDM mapped version of the Pakistan database.

	Table	Number of records mapped
1	LOCATION	357
2	CARE_SITE	2513
3	PERSON	349,879
4	PROVIDER	6,508
5	VISIT_OCCURRENCE	568,026
6	OBSERVATION_PERIOD	349,879
7	CONDITION_OCCURRENCE	405,475
8	MEASUREMENT (Vital Sign/ Lab Results)	33,984,672
9	PROCEDURE_OCCURRENCE	2,173,570
10	DRUG_EXPOSURE	1,003,744
11	SPECIMEN	2,554,310
12	DEATH	1,537
13	OBSERVATION	635,300
14	DEVICE_EXPOSURE	419,698
15	NOTE	8,042,865

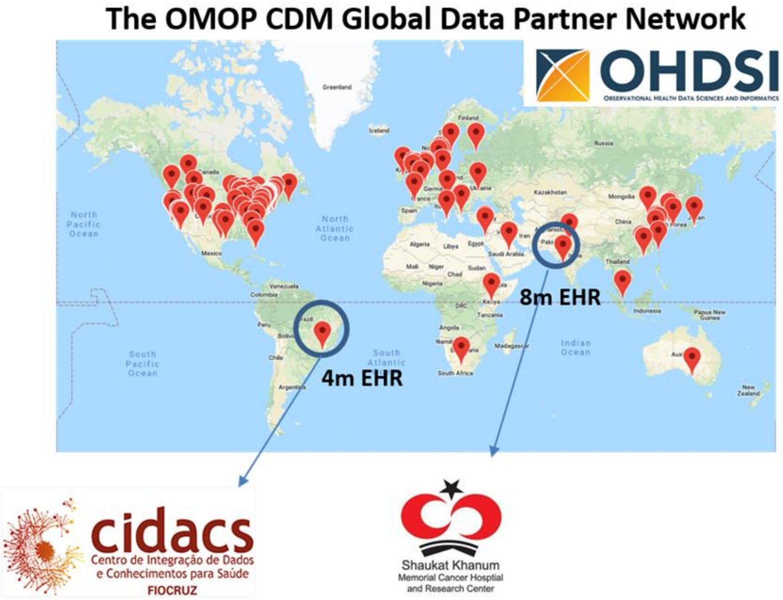

Figure S1: The Observational Health Data Sciences and Informatics (OHDSI) Covid-19 network of databases mapped to the OMOP common data model.

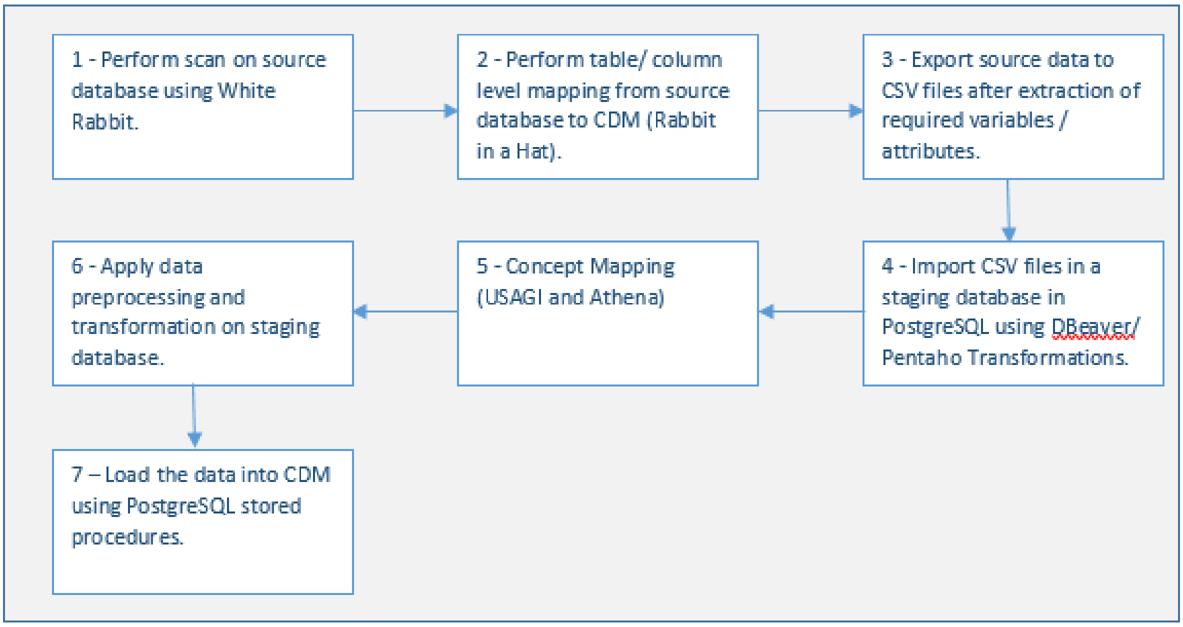

Figure S2: ETL harmonisation steps for the SKMCH&RC (Pakistan) dataset.

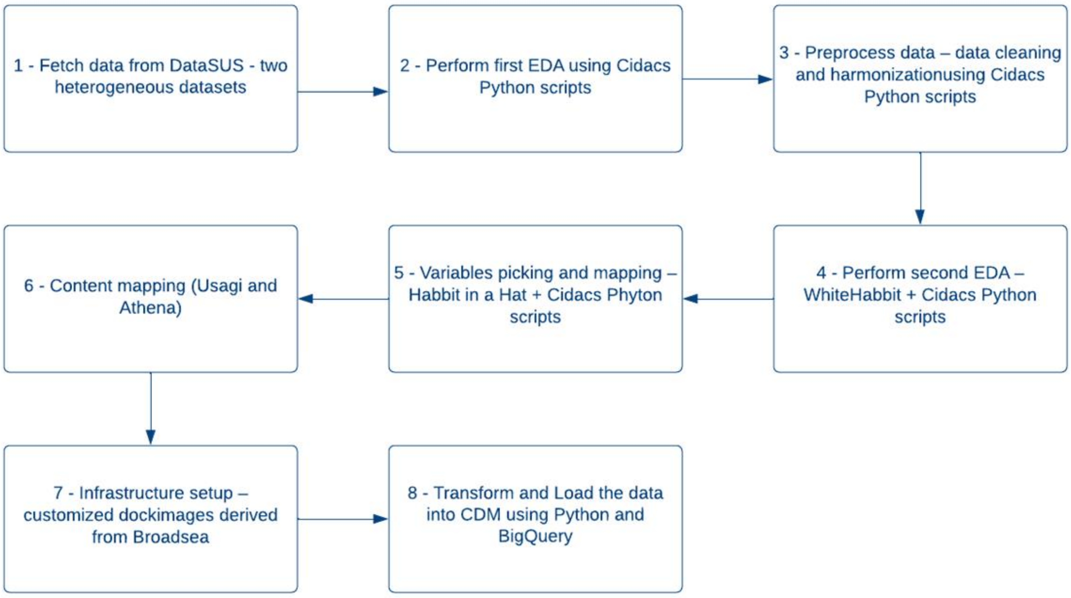

Figure S3: ETL harmonisation steps for the CIDACS-FIOCRUZ (Brazil) COVID-19 dataset.

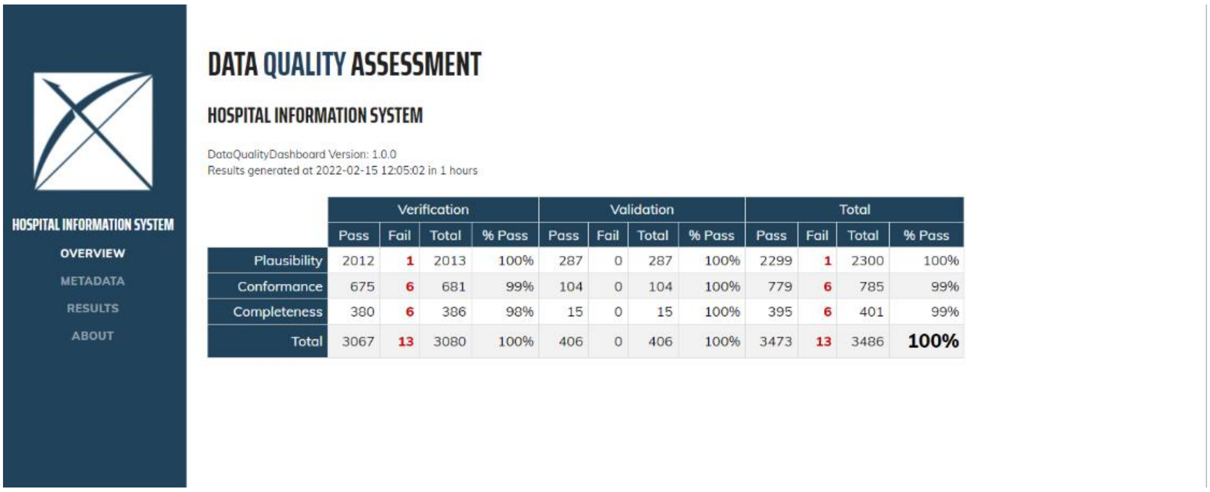

	Verification				Validation				Total			
	Pass	Fail	Total	% Pass	Pass	Fail	Total	% Pass	Pass	Fail	Total	% Pass
Plausibility	2012	1	2013	100%	287	0	287	100%	2299	1	2300	100%
Conformance	675	6	681	99%	104	0	104	100%	779	6	785	99%
Completeness	380	6	386	98%	15	0	15	100%	395	6	401	99%
Total	3067	13	3080	100%	406	0	406	100%	3473	13	3486	100%

Figure S4: Results for data quality checks for SKMHR&C COVID-19 database performed using Data Quality Dashboard (version 1.0.0).

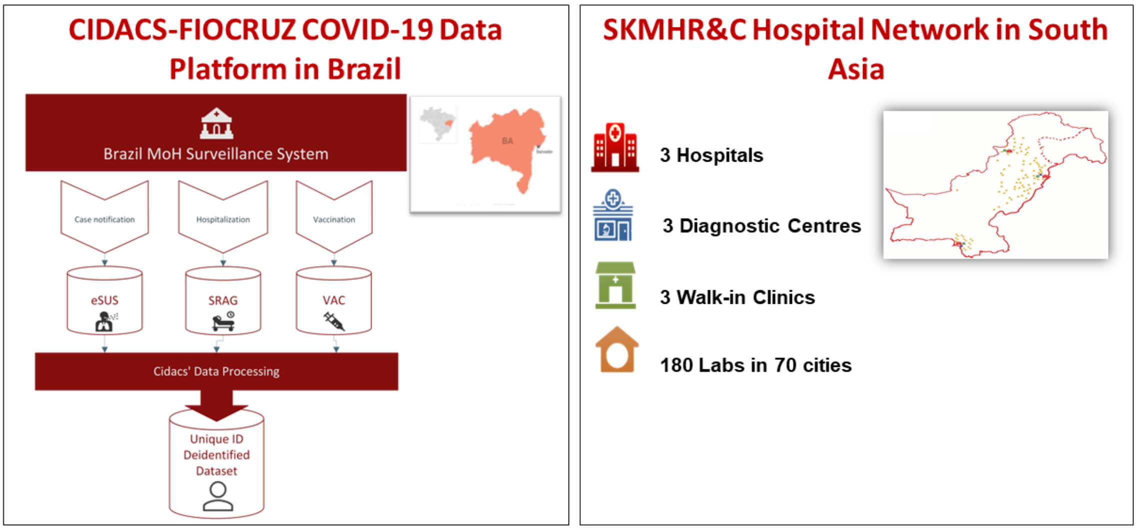

Figure S5. Healthcare settings contributing to the generation of the Brazil and Pakistan data sources.

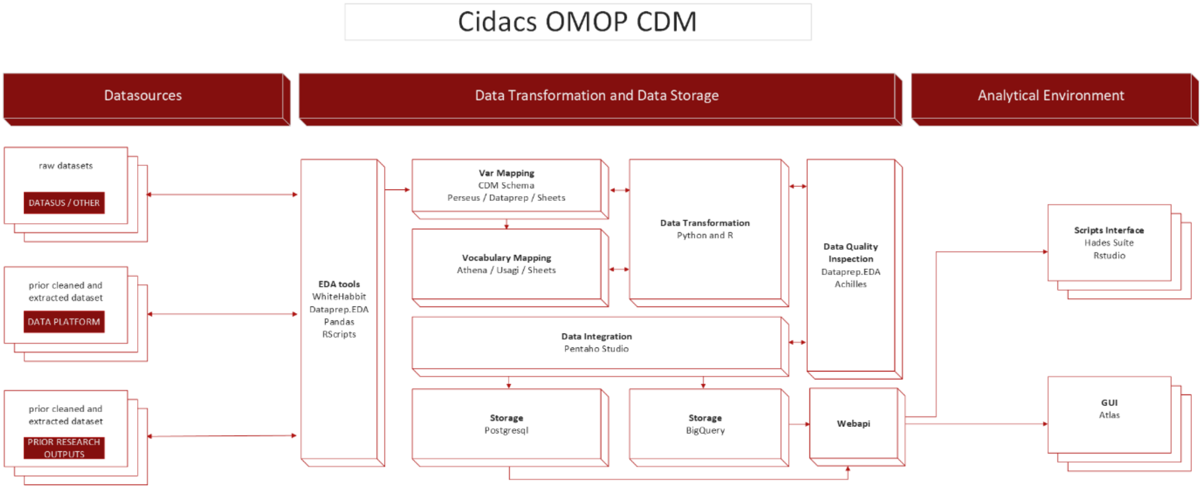

Figure S6: CIDACS-FIOCRUZ (Brazil) data integration platform.
